# Complete mitochondrial genome of the olive weevil, *Dyscerus cribripennis* (Coleoptera: Curculionidae)

**DOI:** 10.1080/23802359.2020.1845580

**Published:** 2021-01-05

**Authors:** Bao-Xin Wang, Zhi-Hang Zhuo, Rui Fang, Hua Yang, Da-Jiang Zhang, Bo-Long Zhang, Li-Yun Sui, Wen-Jian Ma, Ming-Fu Yang, Wei Yang

**Affiliations:** a Key Laboratory of Ecological Forestry Engineering of Sichuan Province, College of Forestry, Sichuan Agricultural University, Chengdu, PR China; b Chengdu Academy of Agricultural and Forestry Sciences, Chengdu, PR China; c College of Life Science, China West Normal University, Nanchong, PR China; d Guangyuan Academy of Forestry Sciences, Guangyuan, PR China

**Keywords:** Olive weevil, *Dyscerus cribripennis*, mitochondrial genome, phylogenetic analysis

## Abstract

The olive weevil *Dyscerus cribripennis* (Coleoptera: Curculionidae) is an uncontrollable noxious insect to *Olea europaea*. The 15,977 bp complete mitochondrial genome of *D. cribripennis* contained 13 protein-coding genes (PCGs), 2 ribosomal RNA genes (rRNAs), 21 transfer RNA genes (tRNAs), and a control region (GenBank accession number MW023069). The *trnI* was not found in the *D. cribripennis* mitogenome. The phylogenetic analysis based on mitogenomes showed that *D. cribripennis* is closed related with *Hylobitelus xiaoi*.

The olive weevil, *Dyscerus cribripennis* Matsumura et Kono (Coleoptera: Curculionidae) is an uncontrollable pest which caused serious damage to *Olea europaea* (Gao et al. 2018; Xiao 2020). The larvae of *D. cribripennis* bore into the stem base of bole and the imagoes gnaw at shoots. Adult specimens of *D. cribripennis* were collected from Lizhou District, Guangyuan City, Sichuan Province, China (N32°23′20′′, E105°39′32′′) in 18 November 2019. The specimen was deposited in the insect specimen room of College of Forestry, Sichuan Agricultural University (voucher no. D018003.2).

The circular mitochondrial genome of *D. cribripennis* was 15,977 bp in length (GenBank accession number MW023069). This mitogenome contained 13 protein-coding genes (PCGs), 2 ribosomal RNA genes (rRNAs), 21 transfer RNA genes (tRNAs), and a control region. The *trnI* was not found in the *D. cribripennis* mitogenome, as observed in *Sympiezomias velatus* (Tang et al. 2017). The gene order and orientation of *D. cribripennis* are identical to the inferred ancestral arrangement of insects (Boore 1999).

The nucleotide composition of the mitogenome was significantly biased (A, G, C, and T was 39.18%, 9.33%, 15.17%, and 36.31%, respectively), with A + T contents of 75.49%. The AT-skew and GC-skew of this genome were 0.038 and −0.238, respectively. Fourteen genes were transcribed on the J-strand, whereas the others were oriented on the N-strand. Gene overlaps were present at 12 gene junctions and involved a total of 94 bp, and the longest overlap (41 bp) existed between *trnH* and *nad4*.

The phylogenetic tree (Zhang et al. 2020) were constructed based on the amino acid sequences of the 13 PCGs from the mitochondrial genomes of 17 Curculionidae species, and *Epicauta ruficeps* was used as an outgroup. The result supported that *D. cribripennis* is closed related with *Hylobitelus xiaoi* (Figure 1).

**Figure 1.**
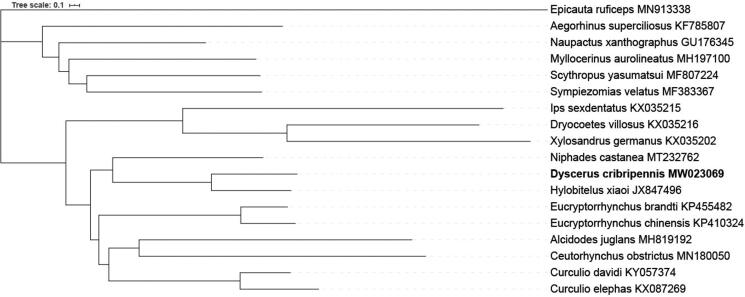
Bayesian Inference phylogenies were inferred using MrBayes 3.2.6 under partition model (2 parallel runs, 2,000,000 generations), in which the initial 25% of sampled data were discarded as burn-in. Bootstrap values were indicated around nodes. GeneBank accession numbers of each species were listed in the tree.

## Date availability statement

The data that support the findings of this study are openly available in the NCBI Genbank database at https://www.ncbi.nlm.nih.gov/genbank/, reference number MW023069.
